# Use of Gallbladder Width Measurement by Computed Tomography in the Diagnosis of Acute Cholecystitis

**DOI:** 10.3390/diagnostics12030721

**Published:** 2022-03-16

**Authors:** Yong Suk Park, Hee Yoon, Soo Yeon Kang, Ik Joon Jo, Sookyoung Woo, Guntak Lee, Jong Eun Park, Taerim Kim, Se Uk Lee, Sung Yeon Hwang, Won Chul Cha, Tae Gun Shin

**Affiliations:** 1 Department of Emergency Medicine, Samsung Medical Center, Sungkyunkwan University School of Medicine, Seoul 06351, Korea; yongsuk.park@samsung.com (Y.S.P.); syrei3.kang@samsung.com (S.Y.K.); ikjoon.jo@samsung.com (I.J.J.); guntak.lee@samsung.com (G.L.); jongeun7.park@samsung.com (J.E.P.); taerimi.kim@samsung.com (T.K.); seuk.lee@samsung.com (S.U.L.); sygood.hwang@samsung.com (S.Y.H.); wc.cha@samsung.com (W.C.C.); taegun.shin@samsung.com (T.G.S.); 2 Biomedical Statistics Center, Data Science Research Institute, Research Institute for Future Medicine, Samsung Medical Center, Seoul 06351, Korea; wsy.woo@samsung.com

**Keywords:** acute cholecystitis, computed tomography, CT, diagnosis, gallbladder

## Abstract

This study aimed to evaluate the diagnostic value of gallbladder width measurement with computed tomography (CT) in patients with acute cholecystitis. This retrospective case–control study was conducted between March 2016 and March 2020 at a tertiary emergency department. Of 310 patients, 254 patients with acute cholecystitis confirmed by surgery were compared with 254 patients diagnosed with other diseases (controls). In the acute cholecystitis group, the number of older patients with underlying illnesses was much higher (64% of men). Upon CT, the median (interquartile range [IQR]) gallbladder width was significantly longer in patients with acute cholecystitis (2.26 [1.82–2.78] cm vs. 3.73 [3.32–4.16] cm, *p* < 0.001). The optimal cut-off value of gallbladder width for differentiating acute cholecystitis was 3.12 cm, showing a sensitivity of 88% and specificity of 86%. In a multivariable analysis using a logistic regression model for diagnosing acute cholecystitis with CT findings (gallbladder width, length, stone, wall thickening, and pericholecystic fluid), a gallbladder width of ≥3.12 cm was significantly meaningful, even when adjusting for other variables (odds ratio 37.9; *p* < 0.001). Therefore, an increase in gallbladder width (≥3.12 cm) measured with CT can be a simple and sensitive diagnostic sign of acute cholecystitis, supporting the underlying pathophysiology of bile outflow obstruction.

## 1. Introduction

Acute cholecystitis is responsible for 3–10% of all cases of abdominal pain and is the most common cause of right upper quadrant pain in the emergency department (ED) [1,2,3,4]. The Tokyo Guideline 2018 recommends ultrasound as the first-choice imaging modality for the diagnosis of acute cholecystitis [5]. This is because of the low invasiveness, widespread availability, convenience of use, and cost-effectiveness of ultrasound. A meta-analysis of ultrasound diagnostic performance for acute cholecystitis reported pooled sensitivities and specificities of 81% (95% confidence interval [CI]: 0.75–0.87) and 83% (95% CI: 0.74–0.89), respectively [6]. However, the diagnostic criteria for ultrasound and its diagnostic yield vary with studies, all of which were had small patient sizes at a single institution. In addition, performing ultrasound is difficult in patients with obesity, gaseous distension, or those who underwent abdominal surgery [7], and there are also tough circumstances where applying ultrasound because of crowded ED or insufficient infrastructure [8].

In recent decades, computed tomography (CT) has become widely used in the ED to diagnose and treat patients with abdominal pain, and it is also frequently employed to diagnose acute cholecystitis [9,10]. CT scans are reported to be more sensitive than ultrasound in some studies and also have advantages in differentially diagnosing other conditions or identifying cholecystitis complications [7,11]. Because cholecystitis requires immediate treatment depending on its severity, it is critical to recognize the common imaging findings and make a timely diagnosis. Characteristic CT findings suggestive of acute cholecystitis include impacted gallstones, distension of the gallbladder lumen, gallbladder wall thickening, and pericholecystic fat infiltration or fluid collection [12]. However, if we focus on the obstruction of bile outflow, which is the most prevalent cause of acute cholecystitis, gallbladder distension can certainly contribute as a fundamental and sensitive imaging finding for acute cholecystitis.

Although the general definition of gallbladder distention is an increase by 8 cm in length and 4 cm in width, clinical data supporting this definition are scarce [13,14]. A recent study found that insufficient gallbladder dilatation by ultrasound, defined as a width of <2.2 cm, is an extremely sensitive indicator for eliminating acute cholecystitis regardless of other ultrasound findings or clinical evidence [15]. However, no study has suggested CT-based diagnostic criteria for gallbladder width. Therefore, this study aimed to evaluate the diagnostic value of characteristic CT findings in patients with acute cholecystitis and, in particular, investigate the diagnostic value of CT-measured gallbladder width compared with clinical and other CT findings. 

## 2. Materials and Methods

### 2.1. Study Design and Setting

This retrospective case–control study aimed to identify the usefulness of gallbladder width measured by CT for the diagnosis of acute cholecystitis in the ED. We searched the electronic medical records of the ED at our tertiary institution in an urban area between March 2016 and March 2020. This study was approved by the Samsung Medical Center Institutional Review Board as an exempt study, and informed consent was waived because of the retrospective nature of clinical and imaging data collection (IRB file number: 2021-03-087-002).

### 2.2. Patient Populations

**Figure 1:**
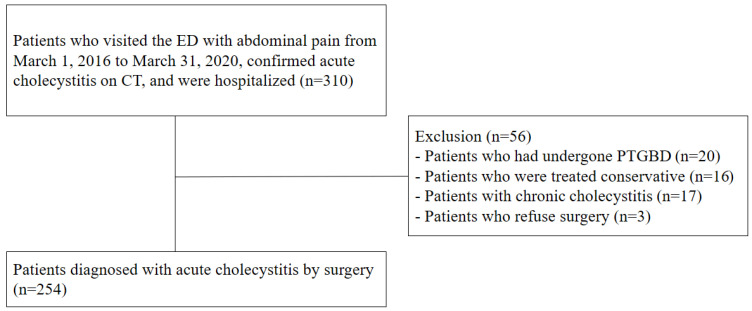
Patient populations with acute cholecystitis. Abbreviations: ED, emergency department; CT, computed tomography; PTGBD, percutaneous transhepatic gallbladder biliary drainage.

During the study period, 310 patients who presented to the ED with right upper quadrant pain were hospitalized after a computed tomography (CT) scan revealed acute cholecystitis. Of these, patients who received percutaneous transhepatic gallbladder biliary drainage (*n* = 20) or conservative treatment (*n* = 16), those diagnosed with chronic cholecystitis (*n* = 17), and those who refused surgery (*n* = 3) were excluded. Therefore, 254 patients who were surgically diagnosed with acute cholecystitis were enrolled in the case group (Figure 1).

A total of 254 consecutive patients who visited the ED for right upper quadrant pain between January and February 2019 and underwent abdominal CT scan were enrolled in the control group. Patients with parenchymal diseases, such as hepatitis or cirrhosis, acute pancreatitis, cholangitis, or biliopancreatic malignancy were excluded. Diagnoses based on medical records included enterocolitis (*n* = 50), cancer-related pain (*n* = 38), gynecologic disease (*n* = 33), ileus (*n* = 33), ureter stone (*n* = 32), appendicitis (*n* = 27), peritonitis (*n* = 9), genitourinary diseases (*n* = 8), hernia (*n* = 5), diverticulitis (*n* = 5), and others (*n* = 14).

### 2.3. CT Imaging Analysis

**Figure 2:**
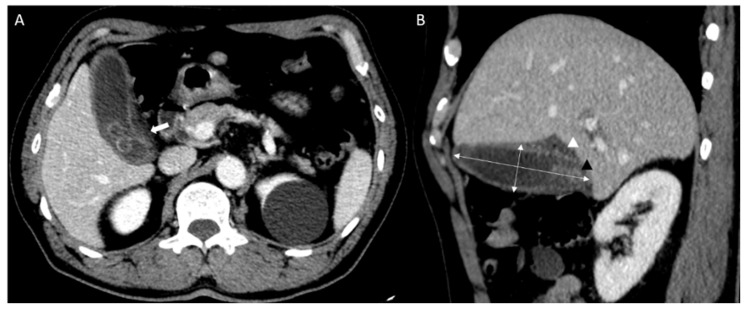
Abdominal computed tomography (CT) findings of acute cholecystitis in a 71-year-old with leukocytosis and right upper quadrant pain ((**A**) axial scan, (**B**) sagittal scan). Abdominal CT results showing gallbladder (GB) distension (double arrow; GB width, 3.36 cm and GB length, 9.34 cm) with impacted GB stone (black triangle). It demonstrates fat infiltration (white arrow) and pericholecystic fluid collection (white triangle).

All CT scans were performed using a commercially available multidetector CT scanner (Discovery CT750 HD; GE Medical Systems, Milwaukee, WI, USA). CT images were retrospectively reviewed by two emergency physicians (P.Y.S. and Y.H.) from the records read by the radiologist. CT images of the cholecystitis and control groups were used to measure (1) gallbladder width, (2) gallbladder length, (3) presence of stones, (4) wall thickness, (5) pericholecystic fluid, and (6) fat infiltration. Gallbladder length was calculated using the maximum length among the axial, coronal, and sagittal planes of the abdominal CT, and the gallbladder width was measured using the greatest length of the outer-to-outer margin of the plane perpendicular to gallbladder length (Figure 2) [16].

### 2.4. Clinical Data Collection

The following data were recorded: patient sex, age, presence of underlying diseases (hypertension, diabetes mellitus, cardiac disease, chronic lung disease, intraperitoneal cancer, and ascites), initial vital signs (blood pressure, respiratory rate, heart rate, and body temperature), laboratory data (white blood cell, bilirubin, liver enzymes, creatinine, C-reactive protein, etc.), and clinical data (pathology, and clinical diagnosis).

### 2.5. Outcomes

The primary endpoint of this study was the difference in the gallbladder width measured on CT in each group. The secondary outcome was the difference in other CT findings, such as gallbladder length, wall thickness, presence of stones, pericholecystic fluid collection, and fat infiltration on CT in each group. In addition, we investigated the optimal cut-off values of gallbladder width for differentiating acute cholecystitis from the control group. It also includes the determination of relevant cholecystitis predictions based on patient demographics, laboratory testing, and CT findings.

### 2.6. Statistical Analysis

Standard descriptive statistics were used to present all data. Continuous variables are presented as medians with interquartile ranges (IQR). Categorical data were presented as numbers and percentages. Logistic regression analysis was used to evaluate the association between gallbladder width and length and acute cholecystitis. Firth’s penalized likelihood method was used to avoid bias in parameter estimates due to rare events for some variables [17]. Using receiver operating characteristic (ROC) curve analysis, the optimal cut-off value was determined by assessing the point closest to (0, 1) (i.e., the upper-left corner of the unit square) on the ROC curve. Multi-collinearity was assessed for variables with a *p*-value of <0.05 using the variance inflation factor (VIF) index. Except for variables with VIF of >4 showing multi-collinearity and values with heavy missing data, a backwards selection in multivariable regression analysis was performed on the association between clinical and CT findings and cholecystitis. The sensitivity, specificity, accuracy, positive predictive value, and negative predictive value of each significant imaging finding were determined. All statistical analyses were performed using the SAS ver. 9.4 software (SAS Institute, Cary, NC, USA) and R 4.1.0 (Vienna, Austria; Available online: http://www.R-project.org/ (accessed on 21 January 2022)).

## 3. Results

The demographics, vital signs, laboratory data, and CT findings of each of the 254 patients in both groups are summarized in Table 1. Patients in the acute cholecystitis group were significantly older than those in the control group (49 [34–61] vs. 65 [56–75]) (median [IQR]), and they were predominantly male (39% vs. 64%). In addition, there were more underlying diseases such as diabetes, hypertension, and heart disease in the acute cholecystitis group. There were no statistical differences in vital signs, except for body temperature. However, the median values of blood laboratory data, such as white blood cell count and total bilirubin, aspartate aminotransferase, alanine aminotransferase, alkaline phosphatase, and C-reactive protein levels, were significantly higher in the cholecystitis group (Table 1).

**Figure 3:**
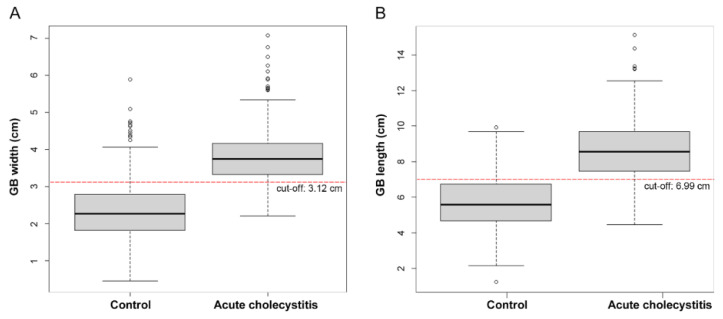
Box plot of acute cholecystitis and control group by gallbladder (GB) width (**A**) and length (**B**).

On CT scans, the median (IQR) gallbladder width was 2.26 (1.82–2.78) cm and 3.73 (3.32–4.16) cm in patients in the control and acute cholecystitis groups, respectively. This was significantly longer in patients with acute cholecystitis (*p* < 0.001). The gallbladder length (8.56 [7.44–9.68] cm) and wall thickness (3.0 [2.3–3.7] mm) (median [IQR]) were also significantly longer and thicker, respectively, and there were more gallstones, fat infiltration, and pericholecystic fluid in the cholecystitis group (Table 1, Figure 3).

**Figure 4:**
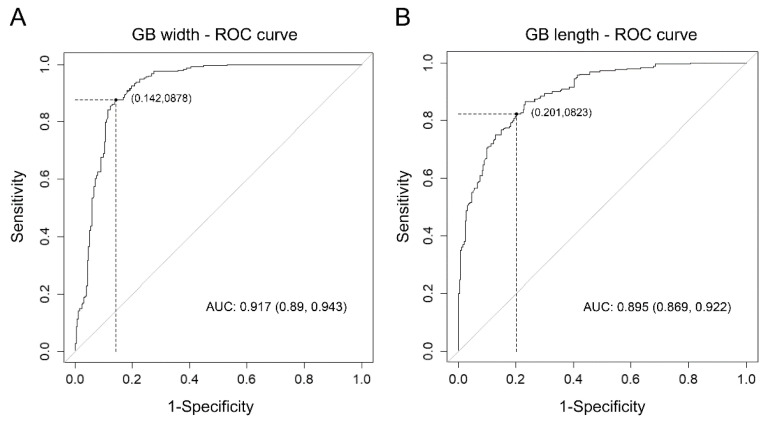
(**A**) The ROC curve for univariable logistic regression analysis of the association between GB width and acute cholecystitis, (**B**) ROC curve for univariable logistic regression analysis of the association between GB length and acute cholecystitis. (Abbreviations: GB, gallbladder; ROC, receiver operating characteristic; AUC, area under the curve.)

The optimal cut-off values of gallbladder width and length for differentiating acute cholecystitis from the control group determined by assessing the ROC curve were 3.12 cm and 6.99 cm, respectively. Applying this cut-off value of gallbladder width yielded the best sensitivity at 87.8% and specificity at 85.8% for diagnosing acute cholecystitis. None of the patients had acute cholecystitis with a gallbladder width of <2.2 cm. When a gallbladder length cut-off of 6.99 cm was applied, the sensitivity was 82.2%, and specificity was 79.9% (Table 2, Figure 4).

In multivariable regression analysis with clinical and CT findings, gallbladder width ≥ 3.12 cm (odds ratio [OR] 13.81; *p* < 0.001), gallbladder length ≥ 6.99 cm (OR 7.24; *p* < 0.001), gallbladder stone (OR 12.25; *p* < 0.001), gallbladder wall thickening (OR 16.57; *p* < 0.001), age (OR 1.03; *p* = 0.03), and total bilirubin (OR 3.03; *p* < 0.001) were significant independent variables for predicting acute cholecystitis (Table 3, Appendix A).

When performing multivariable regression analysis for diagnosing acute cholecystitis only with CT findings (excluding fat infiltration variable with multi-collinearity), the gallbladder width of ≥3.12 cm was significant even when adjusting the GB length, GB stone, GB wall thickness, and pericholecystic fluid (OR 37.9; *p* < 0.001) (Table 4).

## 4. Discussion

Abdominal pain continues to pose a diagnostic challenge to emergency clinicians, as differential diagnoses range from benign to life-threatening conditions [18,19,20]. Some patients with cholecystitis present to the ED with ambiguous symptoms, such as chest discomfort, diffuse abdominal pain, anorexia, high fever, and back pain [21]. A CT scan cannot evaluate the sonographic Murphy’s sign and may not show the gallstone due to the nature of the stone. Nevertheless, CT is widely used because it is more effective in the differential diagnosis and detection of accompanying complications for cholecystitis [9]. In this study, we evaluated the diagnostic yield of CT performed in the ED for patients with acute cholecystitis. The diagnostic accuracy of gallbladder width (≥3.12 cm) and gallbladder length (≥6.99 cm), indicating gallbladder distension, was high at 87% and 81%, respectively. In particular, in the multivariable regression analysis for predicting acute cholecystitis based on CT findings, gallbladder width (≥3.12 cm) showed a high OR of 37.9, even when adjusting for other variables. As a result, a simple CT measurement of gallbladder width (≥3.12 cm) was found to be very sensitive diagnostic evidence of acute cholecystitis, supporting the underlying pathophysiology of bile outflow obstruction.

Several studies compared the diagnostic accuracies of different imaging modalities for the detection of acute cholecystitis. In a comprehensive meta-analysis of 57 studies evaluating the imaging diagnostic performance of acute cholecystitis in 2012, the sensitivity of cholescintigraphy (96%; 95% CI: 94–97%) was significantly higher than that of ultrasound (81%; 95% CI: 75–87%) and magnetic resonance imaging (85%; 95% CI: 66–95%) [6]. In that meta-analysis, only one study on CT was included, and the reported sensitivity was 94% (95% CI: 73–99%) with a specificity of 59% (95% CI: 42–74%). In a 2018 study of 42 patients at the Veterans Administration Hospital, CT was significantly more sensitive to the diagnosis of acute cholecystitis than ultrasound (85% vs. 68%) [7]. However, even in this study, the number of subjects was small, and the diagnostic yield of each characteristic CT finding could not be compared. In a situation where the accuracy of CT scans for cholecystitis diagnosis is under-evaluated, it is meaningful to reveal the high accuracy of CT diagnosis in a relatively large number of surgically confirmed patients. Above all, the sensitivity and specificity of a single finding with increased GB width rather than the complete CT finding are somewhat greater than previously reported ultrasonic diagnostic accuracy, making it simple to use in clinical practice.

Although the accepted definition of gallbladder distention (8 cm in length and 4 cm in width) is reported in the literature [13,14], there is a paucity of data supporting this definition. According to an ultrasound study by Martinez et al., 87% of 24 cholecystitis patients had a gallbladder width of ≥4 cm, whereas 96% of 30 fasted controls had a gallbladder width of <4 cm [22]. In a study in 2010 comparing CT-measured fasting gallbladder volume, patients with acute cholecystitis had a significantly larger gallbladder volume [23]. However, the three-dimensional gallbladder volume was calculated in such a complicated way with many correction factors, and they could not offer a cut-off value for clinical use. Although CT provides detailed images of the internal organs in the axial, sagittal, and coronal sections, there may be inherent difficulties in accurately determining the lengths along the gallbladder axis [24]. However, the gallbladder width is thought to be a useful indicator of gallbladder dilatation because it is relatively less affected by measurement errors along the axis. In our study, the median (IQR) gallbladder width and length in the acute cholecystitis group were 3.73 (3.32–4.16) cm and 8.56 (7.44–9.68), which were significantly longer than those in the control group. It can be used as a basis for the value of 4 × 8 cm, which is presented as the gallbladder dilatation value for acute cholecystitis.

In Shaish’s study, gallbladder dilatation of <2.2 cm in the gallbladder width measured by ultrasound was presented as a clinically meaningful cut-off value to rule out acute cholecystitis [15]. Similarly, there were no patients with acute cholecystitis with a gallbladder width of <2.2 cm as measured by CT in this study. However, when the cut-off value of 3.12 cm was applied to the gallbladder width, 31 patients with cholecystitis (12%) had a value smaller than the cut-off gallbladder width. Among them, 16 (52%) had gangrenous gallbladder or empyema with gallbladder perforation, and 10 (32%) had early cholecystitis. Gallbladder distension may not be severe in patients with gallbladder perforation, and early acute cholecystitis is generally not supposed to present with all the major CT findings of acute cholecystitis. There are several studies on CT images commonly seen in early acute cholecystitis, such as the tensile gallbladder fundus sign (resistance of the gallbladder fundus to flattening by the anterior abdominal wall) [25], gallbladder bed hyperemia (pericholecystic hyperenhancement of the liver parenchyma surrounding the gallbladder on arterial phase) [26], high density of the gallbladder wall on unenhanced CT [27], and Pope’s hat sign (a crescent-like low-density stripe between the hepatic sidewall of the gallbladder and the surrounding liver parenchyma) [28]. Since these minor features were proven to be helpful for acute cholecystitis diagnosis, CT may be a more beneficial imaging tool for diagnosing early-stage patients.

In contrast, 11 patients in the control group had a gallbladder width larger than the cut-off value. Among them, 10 patients had intra-abdominal cancer with peritoneal seeding or liver metastasis, and one patient had ileus. Although gallbladder distension related to peritoneal seeding is unknown, anatomical compartmentalization of the peritoneum by mesenteric attachment determines the distribution and flow of body fluids in the abdomen and pelvis [29,30], and bile flow may also be affected. In addition, gallbladder size can increase with fasting time [31], which may have influenced the outcome.

Acute acalculous cholecystitis, another form of cholecystitis, is an inflammatory disease of the gallbladder without evidence of gallstones or obstruction of cystic ducts. Approximately 2–15% of cases of cholecystitis are acalculous and usually occur in very sick hospitalized patients [4,32]. The exact causal mechanism is not clear, but it is associated with an underlying medical condition or clinical trauma, such as major burns, end-stage renal disease, post-hemorrhagic shock resuscitation, surgery, multiple trauma, or leukemia, that can produce systemic inflammation [4]. In patients with acalculous cholecystitis, gallbladder distension was also observed, although gallstones do not obstruct the bile duct, and it is thought to be induced by reduced blood flow to the gallbladder (ischemia), infectious disease, or lack of gallbladder stimulation (not eating), causing biliary stasis (bile immobility) [33,34,35]. As a result, CT findings of a similar aspect appear in both calculous and acalculous cholecystitis [36]. 

In this study, there were more men (61%) in the cholecystitis group, with a higher median age (65 years). In multivariable regression analysis, age was a significant independent variable for predicting acute cholecystitis (OR, 1.03; *p* = 0.03). The cholecystitis group also had considerably higher rates of hypertension; diabetes; and cardiac, lung, and renal diseases. These findings are consistent with the demographic features of cholecystitis patients found in the study by Nikfarjam et al., in which male patients with acute cholecystitis were older, had more comorbidities, and were more likely to have gangrenous cholecystitis than female patients [37]. Lein and Huang also insisted that male sex and age > 60 years are risk factors for acute cholecystitis [38]. Moreover, as our institution is a tertiary emergency medical center, patients without underlying diseases and complications are frequently transferred to secondary hospitals, while patients with severe disease are hospitalized for surgery; this may have contributed to the predominant male sex and age > 60 years.

### Limitations

Our study has several limitations. First, as this was a retrospective study conducted at a single institution, it may have been influenced by selection bias. Only individuals with surgically confirmed acute cholecystitis after a CT scan in the ED were included in the study. Patients who had been transferred to another hospital or who received conservative treatment were not included in the study. Consequently, this may have resulted in variations in sensitivity. Second, there is also the possibility of bias and measurement errors because CT was evaluated by unblinded emergency physicians. However, the data were compiled based on reports by the radiologist, and if the variable was not described in the reading, the data were collected based on the agreement between the two physicians. Third, the cholecystitis and control groups had varied demographic characteristics in terms of age, sex, and underlying conditions, which could have influenced the outcome. However, these findings are in line with those of previous studies showing the characteristics of patients with cholecystitis. Fourth, although gallbladder size may increase with fasting time, we were unable to control the fasting time of the patients visiting the ED, which may have influenced the outcome. Finally, we did not compare the accuracy of diagnosis with imaging modalities other than CT; therefore, we cannot explain the accuracy of CT compared with others.

## 5. Conclusions

An increase in gallbladder width measured by CT (≥3.12 cm) was found to be 87% accurate in the diagnosis of acute cholecystitis, which can serve as a simple and sensitive diagnostic marker supporting the underlying pathophysiology of bile outflow obstruction.

## Figures and Tables

**Table 1:** Patient characteristics, laboratory data, and computed tomography findings.

	Control (*n* = 254)	Cholecystitis (*n* = 254)	*p*-Value
Demographics			
Sex—male	98 (39)	162 (64)	<0.001
Age (year)	49 (34–61)	65 (56–75)	<0.001
Hypertension	41 (16)	104 (41)	<0.001
Diabetes mellitus	19 (7)	65 (26)	<0.001
Cardiac disease	15 (6)	45 (18)	<0.001
Chronic lung disease	2 (1)	16 (6)	0.001
Chronic renal disease	1 (1)	7 (3)	0.037
Intraperitoneal cancer	66 (26)	41 (16)	0.005
Ascites	58 (23)	54 (21)	0.182
Vital signs			
Systolic blood pressure (mmHg)	128 (115–147)	133 (118–151)	0.139
Diastolic blood pressure (mmHg)	79 (70–90)	79 (68–89)	0.469
Heart rate (/min)	87 (77–99)	85 (75–99)	0.731
Respiratory rate (/min)	18 (16–20)	18 (18–20)	0.066
Body temperature (°C)	36.8 (36.4–37.2)	37 (36.5–37.5)	<0.001
Laboratory data			
WBC (×10³/μL)	9.2 (6.8–12.2)	11.5 (8.5–14.7)	<0.001
Total bilirubin (mg/dL) (*n* = 252/254)	0.5 (0.4–0.7)	1.0 (0.7–1.8)	<0.001
AST (U/L) (*n* = 253/254)	21 (18–28)	30 (22–60)	<0.001
AST (U/L) (*n* = 253/254)	15 (11–25)	28 (17–99)	<0.001
ALP (U/L) (*n* = 114/225)	68 (55–89)	93 (69–134)	0.006
PT (INR) (*n* = 249/253)	1.0 (0.9–1.0)	1.0 (1.0–1.1)	<0.001
C—reactive protein (mg/dL) (*n* = 252/253)	0.5 (0.1–2.8)	5.3 (0.6–16.8)	<0.001
Procalcitonin (ng/mL) (*n* = 39/59)	0.1 (0–0.3)	0.6 (0.1–5.6)	0.216
Creatinine (mg/dL) (*n* = 253/254)	0.8 (0.6–0.9)	0.9 (0.7–1.1)	0.203
Albumin (g/dL) (*n* = 251/253)	4.5 (4.2–4.8)	4.3 (3.8–4.6)	<0.001
Protein (g/dL) (*n* = 250/252)	7.3 (6.9–7.6)	6.9 (6.5–7.4)	<0.001
CT findings			
GB width (cm)	2.26 (1.82–2.78)	3.73 (3.32–4.16)	<0.001
GB length (cm)	5.58 (4.68–6.73)	8.56 (7.44–9.68)	<0.001
GB wall thickness (mm)	1.7 (1.3–2.0)	3.0 (2.3–3.7)	<0.001
GB stone	8 (3)	146 (57)	<0.001
Fat infiltration	0	192 (76)	N/A
Pericholecystic fluid collection	2 (1)	178 (70)	<0.001

The data were described using the median (interquartile range) and number (percentage) of observations. Abbreviations: WBC, white blood cell; AST, aspartate aminotransferase; ALT, alanine aminotransferase; ALP, alkaline phosphatase; PT (INR), prothrombin time international normalized ratio; GB, gallbladder; N/A, not applicable.

**Table 2:** Univariable logistic regression analysis of gallbladder (GB) width and length and acute cholecystitis.

	GB Width			GB Length		
	Estimate	95% CI		Estimate	95% CI	
Cut-off (cm)	3.12			6.99		
Sensitivity	0.8780	0.8313	0.9155	0.8228	0.7702	0.8677
Specificity	0.8583	0.8092	0.8987	0.7992	0.7446	0.8467
PPV	0.8610	0.8127	0.9032	0.8038	0.7501	0.8527
NPV	0.8755	0.8281	0.9115	0.8185	0.7650	0.8622
Accuracy	0.8681	0.8356	0.8063	0.8110	0.7742	0.8442

Abbreviations: CI, confidence interval; PPV, positive predictive value; NPV, negative predictive value.

**Table 3:** Multivariable analysis for CT findings and clinical data affecting acute cholecystitis.

Parameter	Estimate	OR	95% CI		*p*-Value
GB width (≥3.12 cm)	2.6254	13.81	5.17	36.89	<0.001
GB length (≥6.99 cm)	1.9801	7.24	2.71	19.38	<0.001
GB stone	2.5051	12.25	3.96	37.87	<0.001
GB wall thickening	2.8078	16.57	7.35	37.37	<0.001
Age	0.0302	1.031	1.01	1.06	0.03
Total bilirubin	1.1095	3.03	1.59	5.78	<0.001

Abbreviations: CT, computed tomography; GB, gallbladder; OR, odds ratio; CI, confidence interval.

**Table 4:** Multivariable analysis for CT findings affecting acute cholecystitis.

Parameter	Estimate	OR	95% CI		*p*-Value
GB width (≥3.12 cm)	3.635	37.90	9.82	146.35	<0.001
GB length (≥6.99 cm)	2.5584	12.92	3.40	49.05	<0.001
GB stone	2.2058	9.08	2.43	33.87	0.001
GB wall thickening (mm)	2.7453	15.57	5.80	41.81	<0.001
Pericholecystic fluid	5.9556	385.92	48.76	>999.99	<0.001

Abbreviations: CT, computed tomography; OR, odds ratio; CI, confidence interval; GB, gallbladder.

## Data Availability

Data related to this study cannot be sent outside because of information security policies in the hospital.

## Appendix A

**Table A1:** Univariable Analysis for CT Findings and Clinical Data Affecting Acute Cholecystitis.

Variables	Estimate	*p*-Value	OR	95% CI	
				Lower	Upper
Sex	−1.0307	<0.0001	0.357	0.249	0.511
Age	0.059	<0.0001	1.061	1.047	1.074
GB width (≥3.12 cm)	3.7742	<0.0001	43.561	26.021	72.923
GB length (≥6.99 cm)	2.9169	<0.0001	18.485	11.846	28.844
GB stone	3.7274	<0.0001	41.569	19.699	87.719
GB wall thickness	2.6722	<0.0001	14.471	9.093	23.032
Peri-cholecystic fluid *	5.4624	<0.0001	235.67	65.679	845.64
Fat infiltration *	7.3562	<0.0001	>999.999	95.721	>999.999
Ascites	−0.2728	0.1327	0.761	0.533	1.086
Hypertension	1.2815	<0.0001	3.602	2.373	5.468
Diabetics mellitus	1.4478	<0.0001	4.254	2.464	7.342
Cardiac disease	1.2327	<0.0001	3.431	1.858	6.334
Chronic lung disease	2.1366	0.0047	8.471	1.927	37.233
Chronic renal disease *	1.9686	0.0664	7.16	0.875	58.563
Intraperitoneal cancer	−0.6009	0.0069	0.548	0.354	0.848
SBP (mmHg)	0.00532	0.1264	1.005	0.999	1.012
DBP (mmHg)	−0.00344	0.5617	0.997	0.985	1.008
HR (/min)	−0.00083	0.8675	0.999	0.989	1.009
RR (/min)	0.1083	0.0219	1.114	1.016	1.223
BT (°C)	0.4097	0.0009	1.506	1.182	1.919
WBC (×10³/μL)	0.1006	<0.0001	1.106	1.064	1.15
Total bilirubin (mg/dL)	1.8417	<0.0001	6.307	3.947	10.079
AST (U/L)	0.0202	<0.0001	1.02	1.011	1.03
ALT (U/L)	0.0218	<0.0001	1.022	1.014	1.031
ALP (U/L)	0.0047	0.0132	1.005	1.001	1.008
CRP (mg/dL)	0.0974	<0.0001	1.102	1.072	1.134
Procalcitonin (ng/mL)	0.0392	0.2115	1.04	0.978	1.106
Creatinine (mg/mL)	0.1953	0.2317	1.216	0.883	1.674
PT (INR) *	5.933	<0.0001	377.274	56.708	>999.999
Albumin (g/dL)	−0.9176	<0.0001	0.399	0.282	0.566
Protein (g/dL)	−0.7839	<0.0001	0.457	0.344	0.607

* Firth logistic regression was used in cases of separability, which often occurs when an event is rare. Abbreviations: GB, gallbladder; SBP, systolic blood pressure; HR, heart rate; RR, respiratory rate; BT, body temperature; WBC, white blood cell; AST, aspartate aminotransferase; ALT, alanine aminotransferase; ALP, alkaline phosphatase; CRP, C-reactive protein; PT (INR), prothrombin time nternational normalized ratio.
